# Polymorphisms rs12998 and rs5780218 in *KiSS1* Suppressor Metastasis Gene in Mexican Patients with Breast Cancer

**DOI:** 10.1155/2015/365845

**Published:** 2015-02-24

**Authors:** Edhit Guadalupe Cruz Quevedo, Gabriela Monserrat Mimendi Aguilar, Luis Anselmo Juárez Aguilar, Susan Andrea Gutierrez Rubio, Silvia Esperanza Flores Martínez, Ingrid Patricia Dávalos Rodríguez, José Sánchez Corona, Martha Isabel Torres Morán, Roberto Carlos Rosales Gómez, María Cristina Morán Moguel

**Affiliations:** ^1^División de Medicina Molecular, Centro de Investigación Biomédica de Occidente, Instituto Mexicano del Seguro Social, Sierra Mojada, No. 800, Colonia Independencia, 44340 Guadalajara, JAL, Mexico; ^2^Centro Universitario de Ciencias de la Salud, Universidad de Guadalajara, Sierra Mojada, No. 950, Colonia Independencia, 44340 Guadalajara, JAL, Mexico; ^3^Laboratorio de Inmunología, Departamento de Fisiología, Centro Universitario de Ciencias de la Salud, Universidad de Guadalajara, Sierra Mojada, No. 950, Colonia Independencia, 44340 Guadalajara, JAL, Mexico; ^4^División de Genética, Centro de Investigación Biomédica de Occidente, Instituto Mexicano del Seguro Social, Sierra Mojada, No. 800, Colonia Independencia, 44340 Guadalajara, JAL, Mexico; ^5^IMAREFI, Centro Universitario de Ciencias Biológicas y Agropecuarias, Universidad de Guadalajara, Camino Ing. Ramón Padilla Sánchez, No. 2100, Nextipac, 44600 Zapopan, JAL, Mexico

## Abstract

*Aims. KiSS1* is a metastasis suppressor gene associated with inhibition of cellular chemotaxis and invasion attenuating the metastasis in melanoma and breast cancer cell lines. Along the *KiSS-1* gene at least 294 SNPs have been described; however the association of these polymorphisms as genetic markers for metastasis in breast cancer studies has not been investigated. Here we describe two simple PCR-RFLPs protocols to identify the rs5780218 (9DelT) and the rs12998 (E20K) *KiSS1* polymorphisms and the allelic, genotypic, and haplotypic frequencies in Mexican general population (GP) and patients with benign breast disease (BBD) or breast cancer (BC). *Results.* The rs5780218 polymorphism was individually associated with breast cancer (*P* = 0.0332) and the rs12998 polymorphism shows statistically significant differences when GP versus case (BC and BBD) groups were compared (*P* < 0.0001). The H1 Haplotype (G/-) occurred more frequently in BC group (0.4256) whereas H2 haplotype (G/T) was the most prevalent in BBD group (0.4674). *Conclusions.* Our data indicated that the rs5780218 polymorphism individually confers susceptibility for development of breast cancer in Mexican population and a possible role as a genetic marker in breast cancer metastasis for H1 haplotype (Wt/variant) in *KiSS1* gene must be analyzed in other populations.

## 1. Introduction

Breast cancer is the most common malignancy among Mexican mestizo women, accounting for 21.2% of all female cancers [1]. Breast cancer metastasis is a leading cause of death in cancer patients rather than as a primary tumor growth.

*KiSS1* is a metastasis suppressor gene located on chromosome 1q32. It is 6151 base pairs in length and has four exons. The first exon is not translated while exons 2 and 3 are coding exons. KiSS-1 can inhibit chemotaxis and invasion by attenuating the metastasis of breast cancer and melanomas [2]. Several studies suggest a possible role for KiSS1 in regulating events after cell-matrix adhesion and cytoskeleton reorganization [2, 3]. KiSS1 expression reduces the metastatic potential by 95% but does not suppress tumorigenicity. It regulates adhesion molecules such as E-cadherin and diminishes MMP9 expression through NF-*κ*B binding inhibition to the promoter [4–6]. The encoded protein shows a 54-amino acid COOH-terminal extreme which participates as a ligand in G protein-coupled receptors in humans [7–9]. Along the gene* KiSS-1* at least 294 SNPs have been described of which 42 correspond to mutations located in untranslated regions (UTR), 30 in exonic and the rest in intronic regions [10].

The aim of this study is to identify the rs5780218 (9DelT) and the rs12998 (E20K)* KiSS1* polymorphisms and the allelic, genotypic, and haplotypic frequencies in Mexican general population (GP) and patients with benign breast disease (BBD) or breast cancer (BC).

The variants in* KiSS1* gene analyzed in this study were selected on the basis of theoretical functional relevance or biological implications. The 9 del T (rs5780218) polymorphism of* KiSS1* gene is located at the −146 position of the 5′UTR of the mRNA transcript and corresponds to the deletion of an A (adenine). The E20K (rs12998) polymorphism is a G→A transition at position 212 of the cDNA which determines the change of Glu-Lys at residue 20 of the protein [10].

So far it is unknown whether these changes are associated with some alteration in the protein function or whether they confer susceptibility to develop metastasis in breast cancer patients.

## 2. Material and Methods

### 2.1. Study Population

We used information from 225 consecutive breast biopsies and 45 mastectomies from the histopathology files of the Pathological Anatomy Unit at Hospital de Especialidades from Centro Médico Nacional from Instituto Mexicano del Seguro Social. The selection criteria for all patients were unrelated persons, age above 18 years, only female, of the same ethnicity (Mexican mestizos) for two previous generations, no family history of breast cancer, and neither radiotherapy or chemotherapy treatment previous to biopsy. Exclusion criteria included duplicated samples, inability to access patients clinical chart, insufficient amounts of sample, or bad quality DNA after extraction. Only 130/265 samples met the criteria for inclusion in this study. The tissue was histologically typed based on standard criteria [11]. Slides from all cases were reviewed to select specific paraffin-embedded tissue blocks containing primarily affected tissue from which genomic DNA was extracted and analyzed previously for other polymorphisms [12].

One hundred and thirty genomic DNA samples were recovered from paraffin-embedded breast tissue (cases) for the present study, numbered and then processed for genotype analysis by another coauthor who has no knowledge of the histopathological diagnosis. After genotyping was complete, cases were distributed in two groups according to pathological assessment into a breast cancer (BC) group and a benign breast disease (BBD) group (Table 1). Data from 90 genomic DNA extracted from blood samples from general population individuals were also included for comparison (GP group); this group comprises male and female nonrelated and healthy adults (Hardy-Weinberg Equilibrium) and an age range of 18–65 years. All individuals belong to the same Mexican mestizos ethnic group.

### 2.2. Genetic Analysis

DNA was extracted from paraffin-embedded tissue and from blood samples using conventional methods [13, 14] and stored at −20°C. Genotypes for both rs12998 and rs5780218 polymorphisms in* KiSS1* gene were determined by PCR-RFLPs protocols implemented by our group.  Table 2 shows the primer sequences and restriction enzymes used. PCR was done in the final volume of 10 *μ*L, adding MgCl_2_ (3.5 mM), dNTPs (2 mM), primers: forward (0.1 *μ*M), reverse (0.1 *μ*M), DNA (10 ng). PCR conditions were performed with an initial denaturation of 4 min at 94°C followed by 30 cycles (94°C 45 sec; 65°C 45 sec; 72°C 1 min) and a final extention of 10 min at 72°C.

**Figure 1:**
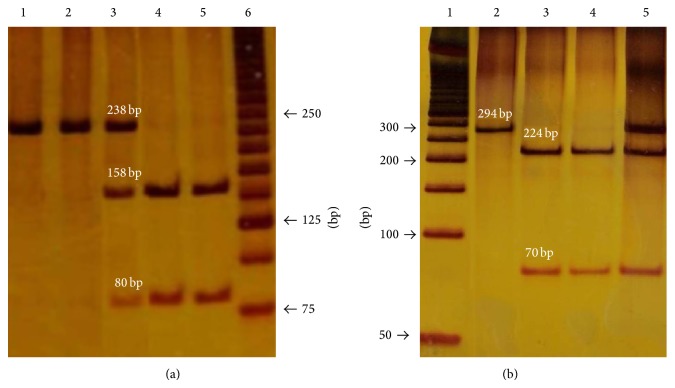
Genotyping of* KiSS1* gene rs12998 (a) and rs5780218 (b) polymorphisms. PCR and hydrolysed products digested with restriction enzymes* Nla* IV or* Sml* I separated on 8% polyacrylamide gels with silver nitrate staining. The molecular weight markers used were a 25 bp ladder and a 50 bp ladder, respectively.

After PCR-RFLPs the 238 bp fragment (rs12998) and the 294 bp fragment (rs5780218) show two bands each of 158 bp and 80 bp and 224 bp and 70 bp, respectively. In the presence of variant alleles the restriction sites are lost in both polymorphisms (Figures 1(a) and 1(b)).

### 2.3. Statistical Analysis

To infer the haplotypes for the groups we used the Haplotype Reconstruction Program *v* = 0.6. Hardy-Weinberg equilibrium was tested on the observed genotypes. All groups were compared using *X* ^2^ and Fisher's exact test when needed; a *P* value of ≤0.05 was considered significant. The strength of any given SNP-cancer association was measured by odds ratio (OR) and its corresponding 95% confidence intervals (CI).

Allelic and genotypic distributions were determined by gene counting and are presented as simple frequencies. Contingency tables and Chi-squared tests were used to compare the allele and genotype frequencies and haplotype association among the BC patients and BBD or healthy individuals as well as to test for the presence of Hardy-Weinberg equilibrium among the genotypes.

## 3. Results

### 3.1. Polymorphisms

A total of 220 DNA samples were analyzed: 78 were from patients that were classified within the BC group whilst 52 were of the BBD group and 90 samples were from the GP group (general population individuals).

Table 3 shows the allelic and genotypic frequencies for rs12998 and rs5780218 polymorphisms in* KiSS1* gene in the three study groups. There is no difference between BC and BBD groups for both polymorphisms; however, when GP was compared* versus* BC or BBD groups there are differences which could be due to the type of tissue (blood versus paraffin-embedded tissue) from which DNA was extracted (Table 3).

### 3.2. Haplotypes

The haplotype construction used the wild type (Wt) or the variant for each allele at rs12998 and rs5780218 polymorphisms. Based on segregation analysis and homozygosity the haplotype phase could be established in 156 chromosomes from BC, 104 chromosomes from BBD, and 180 chromosomes from GP groups. The four possible haplotypes (rs12998: allele 1, rs5780218: allele 2) are H1: Wt/variant (G/-); H2: Wt/Wt (G/A); H3: variant/variant (A/-); and H4 variant/Wt (A/A). All of them were identified in BC and BBD groups, while only three haplotypes were found in the GP group. The haplotype frequencies are shown in Table 4. The comparison between BBD and GP group showed no differences for neither haplotype H1 (Wt/Variant) nor H2 (Wt/Wt). However, the comparative analysis of haplotypes between BC and BBD groups showed significant differences (*P* = 0.0001, OR = 3.32) as well as the BC or BBD groups versus GP (*P* = 0, OR = 3).

## 4. Discussion

In* KiSS1* gene at least 294 SNPs have been identified [10], none previously associated with breast cancer. The rs12998 and rs5780218 SNPs have not been reported previously in relating to cancer studies. In this association study, individuals from a Mexican general population and patients with benign breast disease or breast cancer were considered. The genotype and allele frequencies of both polymorphisms were similar in the studied groups (*P* > 0.05), suggesting that these polymorphisms individually do not confer susceptibility to the development of breast cancer in our population. Furthermore, information contained in the clinical history of some patients from BC group allowed us to identify breast cancer patients with or without metastasis (16 and 13, resp.). When the allele and genotype frequencies were analyzed again for both polymorphisms no significant differences between groups were observed (*P* > 0.5; data not shown). The small sample size has limitation whereby in this study we can only describe these findings without a clear conclusion.

We found differences by comparing the results of rs12998 polymorphism between the case group (BC and BBD) and the general population group (*P* = 0.0000) and between BC* and* GP groups for rs5780218 polymorphism (*P* = 0.0332). However, it is necessary to consider that the biological sample type from which genomic DNA was obtained was different in each group: paraffin-embedded tissue and peripheral blood. So in general population individuals we identified constitutive genotypes of both polymorphisms, while the genotypes in the case group (BC and BBD) were identified in specific tissue (breast) with or without cancer. Loss of heterozygosity (LOH) is the loss of one allele at a specific* locus*, caused by a deletion mutation, or loss of a chromosome from a chromosome pair, resulting in abnormal hemizygosity [15]. It is detected when heterozygous markers for a* locus* appear monomorphic because one of the alleles was deleted. Moreover, in several studies in which polymorphisms in cancer related genes (oncogenes or tumor suppressor genes) have been analyzed, LOH is reported when a discordance between germinal and tumoral genotypes is observed (DNA obtained from peripheral blood and tumor tissue) [15]. Paraffin tissue blocks analyzed in this study were obtained in the 2002–2005 period [11]; therefore we did not have access to peripheral blood samples from these patients and we did not identify the constitutive genotypes in order to establish the possible loss of heterozygosity in rs12998 or rs5780218 polymorphisms that could explain the differences observed between genotypes by type of biological sample.

The haplotype distribution between BC, BBD, and GP groups shows that the H1 haplotype frequency is significantly higher in BC (0.4256) and particularly among patients with breast cancer and metastasis (0.5101), than in BC NM, BBD, or GP groups (Table 3); therefore H1 haplotype might be considered as a possible risk haplotype for breast cancer and metastasis in our population, which had not been described before. However it is necessary to consider the main limitations of this study, as the small sample size. However, even with this limitation, our study is the first analysis of the usefulness of these polymorphisms as potential genetic risk markers for breast cancer, and it was possible to establish the allele, genotype and haplotype frequencies in Mexican population. Moreover it was also limiting to not know the germline genotypes in the study subjects, since we only know the genotype present in the tissue from which DNA was obtained and therefore it is not possible to analyze the loss of heterozygosity in these polymorphic markers. Finally, the information in the clinical charts or medical records was limited or poor in several cases of selected patients.

Moreover, we do not know the biological significance of these polymorphisms in cancer cells, and there is no information about other gene variants described in* KiSS1*. Kisspeptin, encoded by* KiSS1* gene was first considered as a tumor metastasis suppressor; however, it plays a major role in regulating the hypothalamic-pituitary-gonadal axis, so many studies have analyzed polymorphisms or haplotypes in* KiSS1 *gene particularly in central precocious puberty. Huijbregts et al. show that polymorphisms in a G-rich sequence located in the 3′UTR, upstream of the transcription end site of* KiSS1* gene, influence its expression level [16, 17]. Likewise, in polymorphisms and gene expression studies it has been shown that* KiSS1* low mRNA expressions correlate with venous invasion, advanced clinical stage, occurrence of metastasis, and recurrence in patients with different types of cancer [18, 19]. In breast cancer, Kiss-1/GPR54 system, estrogen-related gene expression profiles, regulation, and transcript isoforms of* KiSS1* gene have been described [20–24] and Xie et al. showed significant relationship between lymph node involvement and absence of Kiss-1 expression in early breast carcinoma patients (*P* = 0.001) [25]; however, there is no information about whether the presence of a polymorphism could be involved in the differential* KiSS1* gene expression. Therefore, it will be necessary to confirm in future studies with a larger sample size, if H1 haplotype described here is a possible risk haplotype for cancer and/or metastasis in breast cancer, to analyze their association with metastasis in other cancer types and analyze mRNA expression levels in their correlation with the presence of polymorphisms to know their possible biological significance.

## Figures and Tables

**Table 1:** Diagnosis and age distribution for the studied BC and BBD groups.

Breast cancer group diagnosis	*n*	Age	*n*
Adenocarcinoma	2	<30	2
Ductal adenocarcinoma	4	30–39	10
Invasive ductal adenocarcinoma	47	40–49	20
Invasive lobular carcinoma	5	50–59	12
Lobular adenocarcinoma	1	60–69	11
Lobular and ductal adenocarcinoma	1	>70	4
Residual adenocarcinoma	3	Not available	6
Adenocarcinoma	2		
Ductal adenocarcinoma	4		

BBD group diagnosis	*n*	Age	*n*

Cyst	1	<30	6
Fibroadenoma	10	30–39	12
Fibrocystic mastopathy	14	40–49	10
Fibrosis	2	50–59	7
Hyperplasia	2	60–69	1
Lipoma	2	>70	4
Negative for malignance	9		

**Table 2:** Primer sequences, PCR conditions, and restriction enzymes (RE) used for genotyping rs12998 and rs5780218 polymorphisms in *KiSS1* gene. AT: annealing temperature.

Polymorphism	Primers sequence	AT (°C)	Amplicon (bp)	RE
rs 12998 A/G	F: 5′-ACT TgC TCA CAT TCC ACA gg-3′ R: 5′-gCA TCT CTC TgC TCT TgC AC-3′	65	238	*Nla *IV

rs 5780218 —/A	F: 5′-CCT TTg CCT gCC Tgg ATg CA-3′ R: 5′-Tgg gCC TgT gCT Tgg AgA Cg-3′	65	294	*Sml* I

**Table 3:** Genotype frequencies for *KiSS1* suppressor metastasis gene rs12998 and rs5780218 polymorphisms in three groups and statistical analysis between groups. BC: breast cancer; BCD: breast cancer disease; GP: general population.

	Genotype frequency									Statistical analysis			
Groups (*n*)	BC (78)			BBD (52)			GP (90)			BC *versus* BBD	BC *versus* GP	BBD *versus* GP	BC + BBD *versus* GP
	**GG**	**GA**	**AA**	**GG**	**GA**	**AA**	**GG**	**GA**	**AA**				
rs12998	0.43	0.47	0.08	0.48	0.46	0.05	0.91	0.08	0	*χ* ^2^ = 0.5660 *P *= 0.7688	*χ* ^2^ = 44.92 ***P*** * * **= 0.0000**	*χ* ^2^ = 33.6018 ***P*** * * **= 0.0000**	*χ* ^2^ = 48.8025 ***P*** * * **= 0.0000**

	**AA**	**A—**	—	**AA**	**A—**	—	**AA**	**A—**	—				

rs5780218	0.26	0.39	0.33	0.32	0.44	0.17	0.38	0.44	0.16	*χ* ^2^ = 4.0800 *P *= 0.1368	*χ* ^2^ = 6.7695 ***P*** * * **= 0.0332**	*χ* ^2^ = 0.5724 *P *= 0.7751	*χ* ^2^ = 3.9609 *P *= 0.1415

**Table 4:** Haplotype distribution in patients with breast cancer (BC), breast cancer with metastasis (BC M), breast cancer without metastasis (BC NM), benign breast disease (BBD), and individuals from Mexican general population (GP). Wt: wild type. Haplotypes: H1–H4.

Group (*n*/chromosomes)	Haplotype rs12998/rs5780218				Statistical analysis			
	H1 Wt/variant	H2 Wt/Wt	H3 Variant/variant	H4 Variant/Wt	BC *versus* BBD	BC *versus* GP	BBD *versus* GP	BCM *versus* BC NM
BC (156)	0.4256	0.2474	0.1064	0.2205	*χ* ^2^ = 21.9537 *P* = 0.0002 **(OR** * * **=** * * **3.32)**	*χ* ^2^ = 70.6430 *P* = 0.0000 **(OR** * * **=** * * **3)**	*χ* ^2^ = 0.5724 *P* = 0.7801	*χ* ^2^ = 10.5813 *P* = **0.0130**
BC M (32)	0.5101	0.1773	0.0836	0.2288				
BC NM (26)	0.1431	0.4337	0.2799	0.1431				
BBD (104)	0.2441	0.4673	0.1789	0.1095				
GP (180)	0.3388	0.6166	0.0444	0				
